# Evaluation of the Effectiveness of Two Automated Room Decontamination Devices Under Real-Life Conditions

**DOI:** 10.3389/fpubh.2021.618263

**Published:** 2021-02-23

**Authors:** Birte Knobling, Gefion Franke, Eva M. Klupp, Cristina Belmar Campos, Johannes K. Knobloch

**Affiliations:** Institute for Medical Microbiology, Virology and Hygiene, University Medical Center Hamburg-Eppendorf, Hamburg, Germany

**Keywords:** automated room disinfection, hydrogen peroxide, ozone, real-life condition, terminal cleaning and disinfection, non-touch room decontamination

## Abstract

To evaluate the effectiveness of automated room decontamination devices, a common aerosolized hydrogen peroxide (aHP) as well as a recent gaseous ozone-based device, which produces the disinfectant reagent without the need of consumables, were tested under real-life conditions. Twenty-two contaminated surfaces were positioned in different areas in a patient room with adjacent bathroom and anteroom. Following the decontamination process bacteria were recovered and reduction factors were calculated after performing quantitative culture. Following the manufactures instructions, the ozone-based device displayed a bactericidal effect (log_10_ > 5), whereas the aHP system failed for a high bacterial burden and achieves only a complete elimination of a realistic bioburden (log_10_ 2). After increasing the exposure time to 30 min, the aHP device also reached a bactericidal effect. Nevertheless, our results indicate, that further research and development is necessary, to get knowledge about toxicity, efficacy and safety by using in complex hospital conditions and achieve meaningful integration in cleaning procedures, to reach positive effects on disinfection performance.

## Introduction

Pathogens associated with common nosocomial infections like methicillin resistant *Staphylococcus aureus*, vancomycin-resistant enterococci or *Clostridioides difficile* can survive on dry surfaces for several weeks to month (1). Furthermore, these pathogens are often detected in patient's environment, if patients are colonized or infected (2–5). Contaminated surfaces might be an important source for transmission and acquisition of healthcare associated pathogens (5–8). This recognition is supported by recent studies, which pointed out an increased risk of acquiring these pathogens with possible subsequent healthcare associated infections, if prior room occupants had already been infected (9–12).

Regular cleaning such as terminal cleaning and disinfection of surfaces, have been implemented in hospitals in the past to reduce the risk of transmission by contact to inanimate surfaces (13, 14). However, various studies have demonstrated that adequate disinfection from routine daily cleaning was not achieved. Using a fluorescence method, Carling et al. showed that only an average of 48% of examined surfaces were cleaned successfully (15). In addition, another study demonstrates a terminal cleaning thoroughness of average 57% for frequently touched surfaces after patients discharge (16).

Routine disinfection depends on several human factors, such as the selection of suitable substances, complete application to all relevant surfaces, compliance with the required exposure time, and correct implementation of cleaning protocols. Moreover, the complex hospital environment contains areas, which are unattainable and difficult to clean (17). Furthermore, unclarified responsibilities for cleaning of special sites such as medical equipment negatively affects cleaning and disinfection success (18).

To achieve more effective results automated room disinfection systems were developed to address vulnerabilities associated with manual cleaning and improve patient safety. In hospital settings automated room disinfection devices could be an additional method of disinfection, to prevent environmental-borne transmission. Currently, aerosolized and vapored hydrogen peroxide, chlorine dioxide and ultraviolet germicidal radiation are disinfectants, which were used for room decontamination (18, 19). Different studies had shown the effectiveness of these agents in experimental settings (18–21). The efficacy of hydrogen peroxide has also been demonstrated in hospital settings, e.g., during outbreak situations but also in routine use (19–21). In contrast, gaseous ozone is not a common reagent, because of the need of permanent moisture to achieve effectiveness (22). Consequently, only a few studies reported using ozone for decontamination but not yet for hospital room decontamination (21, 23).

In our study, the disinfection performance of a recently developed, fully automated system for generating ozone from atmospheric oxygen in combination with an integrated nebulizer for controlled increase of room humidity, was compared with a commercial nebulizer for generation of aerosolized hydrogen peroxide (aHP) under realistic conditions.

## Materials and Methods

**Figure 1:**
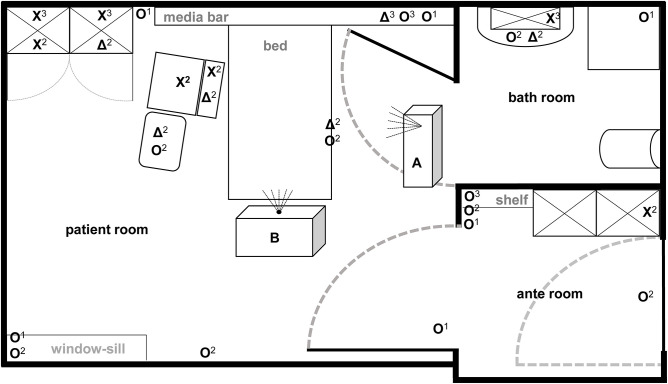
Illustration of test room with depiction of target positions and location of automated room decontamination devices. Both test devices (A: Sentinel H_2_O_2_-Fogger; B: STERISAFE^TM^ Pro Ozone) were positioned at different spots in patient room according to manufactures instructions. The different symbols [O: high contaminated surfaces (HCS); X: HCS into furnishings; Δ: secondary contaminated surfaces (SCS)] represent the kind of contaminated surface used at particular positions. In addition, superscript numbers display the position heights into the room (1 on the ground; 2 middle level; 3 at the top).

The efficacy of aHP and ozone-based devices for automated room disinfection were evaluated in a typical patient room (31.89 m^2^) with adjacent bathroom (6.63 m^2^) and anteroom (7.11 m^2^) as displayed in Figure 1. Aerosolized hydrogen peroxide (aHP) was produced by the Sentinel H_2_O_2_ Fogger system (IC Solutions Leipzig, Germany) and ozone as well as the required humidity were generated by the STERISAFE^TM^ Pro system (STERISAFE Pro version 1.0, STERISAFE ApS, Ole Maaløe's vej 5, DK – 2200 Copenhagen). The STERISAFE^TM^ Pro has an integrated measuring device to monitor and document the ozone concentration as well as humidity. A successful disinfection cycle is only confirmed, if the permissible limit value is exceeded. In addition, at the end of disinfection procedure an active purification phase is included to remove existing ozone by degradation to oxygen and removal of fine dust by filtration.

To prepare standardized contaminated surfaces a suspension of *E. faecium* ATCC 6057 with 5.0 × 10^7^-1.2 × 10^8^ colony forming units (cfu)/mL was produced. 20 μL of this suspension was dried on ceramic tiles (5 × 5 cm, #3709PN00, Villeroy&Boch, Mettlach, Germany) to generate high and primary contaminated surfaces (HCS & PCS). The high contaminated surfaces serve to prove a bactericidal reduction capacity of log10 > 5 (24). Furthermore, surfaces with low contamination were generated by a touch transfer assay, to demonstrate efficacy against a realistic bioburden (25). In brief, dried *E. faecium* was picked up by touching the PCS with one finger covered with a sterile cotton glove after moistening on Columbia Agar with Sheep Blood (COLS+, OXOID Deutschland GmbH, Wesel, Germany) and bacteria were transferred to another sterile ceramic tile to produce the secondary contaminated surface (SCS). Only SCS that met an initial surface load of 5 × 10^2^-5 × 10^3^ cfu were included in the final analysis.

The HCS were placed at 22 certain positions locations in the complex room structure to represent both vertical and horizontal surfaces in different heights and positions [close to patients (*n* = 4); distant from patients (*n* = 10); bathroom (*n* = 3); anteroom (*n* = 5)]. Four SCS were placed close to the patient area, while two were positioned in furnishings within the patient room and bathroom (Figure 1). Also, one HCS and one SCS were placed outside the test room as controls.

Both systems for automatic room disinfection were investigated at least in four independent experiments with identical placement of contaminated surfaces. The disinfection devices were employed according to manufactures instructions. For the ozone-based device, a normal cycle with 70–80 ppm ozone concentration was applied for a holding time of 15 min and 80–90% relative humidity. For the aHP unit a fogging time of 20 min was used in accordance with the manufacturer's specifications depending on the volume of the test room. After insufficient decontamination performance according to manufactures instructions (*n* = 3), we increased fogging time to 30 min (*n* = 4). Before application, doors and ventilation diffusers as well as smoke detectors were sealed.

After each decontamination process bacteria were recovered from both, treated and untreated ceramic tiles, by using flocked nylon swabs (eSwab^TM^ Standard, Copan; Brescia, Italien). After moistening the swab with transport medium, the ceramic tiles were wiped in horizontal, vertical and diagonal direction while the swab was rotated continuously. The bacteria were eluted into the transport medium by vortexing for 30 s and subsequently quantitative cultures were performed in double determination (detection limit 5 cfu/25 cm^2^) by spreading 100 μl of the Amies Medium on COLS+, using a Drigalski spatula. Agar plates were incubated for 18–24 h at 37°C. The reduction factors were calculated by subtracting the log10 of the control and the log10 after disinfection. Statistical analysis was performed in R (26) by using a Bonferroni corrected pairwise *t*-test.

## Results

**Figure 2:**
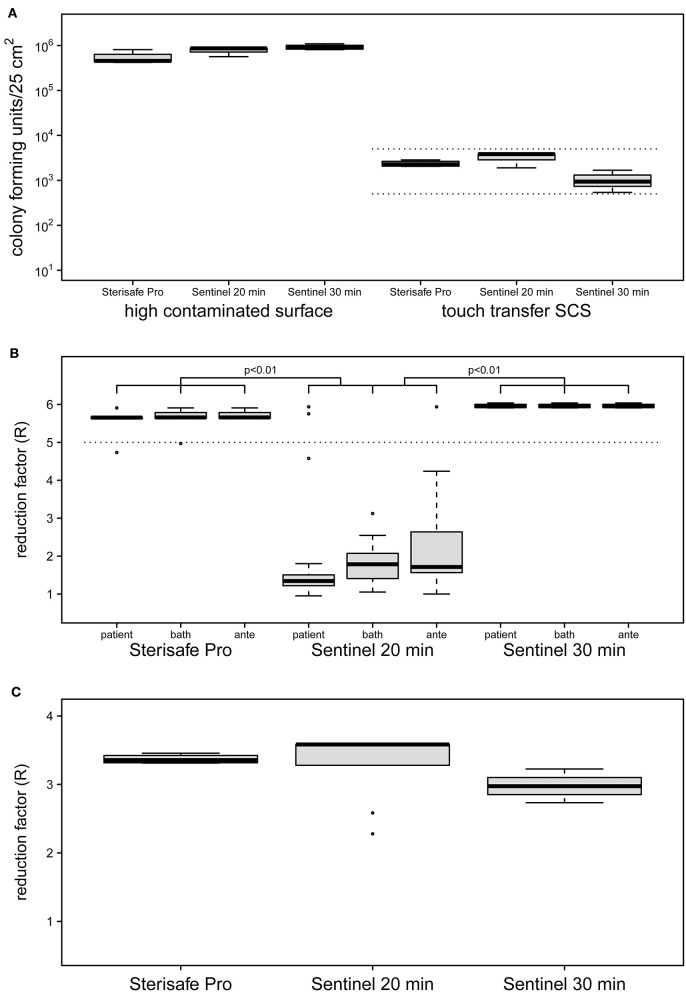
Initial contamination of HCS and SCS surfaces **(A)** and reduction factors achieved by two different automated room decontamination devices **(B,C)**. The distribution of contamination used for the experiments **(A)** are shown separately as boxplot for the HCS and the SCS for the equipment and processes performed. Only SCS that met an initial surface load of 5 × 10^2^-5 × 10^3^ cfu were included (dashed lines). The effectiveness of the disinfection processes was determined in several independent experiments with quantification of recoverable bacterial load in duplicate (STERISAFE^TM^ Pro [*n* = 4], Sentinel 20 min [*n* = 3], Sentinel 30 min [*n* = 4]). The distribution of the calculated reduction factors for the HCS **(B)** and the SCS **(C)** is shown separately as a boxplot for the patient room, the bathroom and the anteroom. Disinfection using STERISAFE^TM^ Pro as well as a 30-min nebulization of H_2_O_2_, achieved the mean reduction rate of 5 log10 (**B**, dashed line) required for a process recognized as disinfection. Both disinfection processes were superior to a 20-min nebulization of H_2_O_2_, as confirmed with the HCS (*p* < 0.01 paired *t*-test with Bonferroni correction). Realistic bacterial contamination was completely eliminated **(C)** for both processes (for aHP regardless of nebulization time). The differences in the distribution of the reduction rates displayed in the boxplots, results exclusively from the differences in the contamination used (**A**, right side).

Evaluation of quantitative cultures of untreated high contaminated surfaces (HCS) showed a mean of 7.4 × 10^5^ cfu/25 cm^2^ for all tested devices (Figure 2A). This surface load is suitable for demonstrating a reduction of >5 log10 and was able to designate a product as a bactericidal disinfectant. The bacterial load of untreated SCS revealed an average of 2.1 × 10^3^ cfu/25 cm^2^ modeling a worst case contamination of frequent touched surfaces (25, 27).

The ozone-based STERISAFE Pro achieved a log10 reduction factor of >5 in all parts of test room, regardless of the placement of the HCS. The total cycle time needed for one decontamination process was ~3 h. The Sentinel H_2_O_2_ Fogger needed about 2 h for one cycle, but did not achieve the reduction rate required for disinfectants, taking into account the manufacturer's instructions. Under these conditions only a mean reduction of 2 log10 was observed. Strikingly, different reduction rates were achieved in the three connected rooms under these conditions. Despite the direction of the device spray into the patient's room, non-significantly lower reduction rates were observed in the patient's room with a mean reduction rate of log10 1.73 compared to the anteroom (mean = 2.31, *p* > 0.05) and the bathroom (mean = 1.86, *p* > 0.05). Because of this, after three experiments the exposure time was increased to 30 min for four additional experiments. After this adaption, the Sentinel H_2_O_2_ Fogger achieved a log10 reduction rate of >5 at all test positions equally (Figure 2B). The reduction factors determined for the Sentinel H_2_O_2_ Fogger at 20 min were significantly less effective compared to the reductions achieved by Sterisafe^TM^ Pro and Sentinel H_2_O_2_ Fogger at 30 min exposure (*p* < 0.01).

Furthermore, no bacteria could be recovered from the SCS after automated room disinfection, regardless of the device used and the amount of nebulized H_2_O_2_. This results in a log10 reduction factor of average >3. The reduction factors of the different devices are only varying in dependence of bacterial load of untreated SCS (Figure 2C). Therefore, a statistical evaluation of these results was omitted.

## Discussion

Manual cleaning is not standardized and often refuses to remove bioburden on frequently touched surfaces, because of different personal-related reasons (17, 18, 22). Therefore, automated room decontamination systems could be a suitable method to enhance the success of cleaning and disinfection processes in hospitals. The efficacy of a procedure, the ease and safety of use, rapid availability and ability to be integrated into routine processes are important for using new standardized procedures for final disinfection (18). Therefore, studies characterizing such devices are essential to ensure effectivity as well as safe operation in hospitals.

The efficacy of the established aerosolized hydrogen peroxide (aHP) process was investigated in comparison to a recently developed fully automated device for disinfection using ozone under conditions as close to reality as possible. All decontamination experiments were carried out in a fully furnished patient room with two adjacent rooms using highly contaminated surfaces according to the European Committee of Standardization (24) as well as surfaces with a realistic bioburden.

Both devices were used according to the manufacturer's specifications. The ozone-based device showed the required bactericidal effect with a reduction of >5 log10, while aHP did not meet the requirement with an average reduction of only 2 log10 for the high contamination. However, a realistic surface contamination, which was modeled with touch transfer, could be completely eliminated by both devices. By extending the fogging time of the aHP device and thus increasing the amount of hydrogen peroxide applied, the full disinfecting efficacy could be achieved.

The obtained results show a bactericidal effectiveness of the ozone device independent of the position in the room. Previous studies demonstrated a reduction of bacteria known to cause hospital-acquired infections by only >3 log10 (28, 29). Here, an ozone concentration of 25 ppm was applied over different exposure times and a relative humidity of 75–95%. However, a reduction of >3 log10 does not meet the requirements for bactericidal disinfection performance (24). Moat et al. assume that an increase in the ozone concentration might led to the achievement of disinfecting efficacy (29). Zoutman et al. showed that a reduction >6 log10 for MRSA could only be achieved at 500 ppm ozone concentration (90 min exposure time) at a relative humidity of 80%, which was produced by a separate humidifier (30). However, enterococci were not sufficiently reduced under these conditions. Only the addition of 1% hydrogen peroxide instead of water nebulized to increase the room humidity resulted in a high efficacy with a 30 min exposure time (30). It was shown that an increased humidity level enhances microbiocidal efficiency (31), our results with full bactericidal efficacy are consistent with these previous data as the new system combines a controlled high relative humidity with a continuous ozone level above 70 ppm for 60 min. These data indicate, that full activity can be reached without the use of additional consumables (e.g., HP) if the process is controlled during the whole disinfection cycle.

The aHP system, only reaches full bactericidal effectiveness after extending the fogging time to 30 min. This is in agreement with observations that aHP shows varying antimicrobial activity depending on the location of contaminated surfaces in the room (17, 32–34). Consistent with Fu et al. (17), we did not observe full efficacy for aHP at all positions when following the manufacturer's instructions, while full efficacy was observed at all positions with an increased amount of nebulized disinfectant. These data indicate that experimental dose finding for an aHP system is required before routine use.

Both systems require a process time of more than 2 h and also require time-consuming preparation (e.g., sealing doors, air diffusers, and smoke detectors with adhesive tape) and therefore cannot be used at all times. Occupational safety aspects are well-taken into account with the STERISAFE^TM^ Pro device, since at the end of the process the active substance is completely degraded and the concentration of ozone prevailing in the room is continuously displayed on a mobile tablet computer and recorded in a standardized manner. In contrast, there is no possibility of monitoring or logging process parameters for the simple aHP nebulizer. For such devices, it is recommended to use additional measurement equipment to verify sufficient concentration and ensure adherence to safety exposure limits afterwards (18). Both principles of action are based on reactive oxygen species that inactivate pathogens, so that sensitive materials could be attacked. However, many medical device manufacturers allow wipe disinfection with hydrogen peroxide, so material compatibility data is available for many materials. Ozone is a highly reactive and corrosive gas (22, 23) and in the future further investigations on material compatibility have to take place.

The following limitations of present study should be noted: The microbiocidal efficiency has only been tested for one pathogen, which is known to be environmentally-resistant. To make a general statement on the effectiveness of such devices in routine use, further investigations with other healthcare associated pathogens such as *S. aureus, C. difficile*, or *Acinetobacter baumannii* should be performed. Furthermore, the influence of soiling and organic load, which have contributed to the reduction in efficacy of automated room decontamination devices in other studies (17, 32), has not yet been included in our study. Additionally, tests should be carried out on various surface materials to assess the impact of different surface texture on disinfection performance.

In conclusion, both ozone-based as well as hydrogen peroxide automated room decontamination systems can achieve bactericidal effectiveness and might have a high potential to improve disinfection performance in hospitals by standardization of the process. The fact that no consumables are needed to generate ozone, could be a decisive advantage especially in pandemic situations like current Covid-19, which, was in part characterized by supply bottlenecks also of disinfectants (35). However, currently unknown aspects of safety and material compatibility as well as long decontamination cycles might restrain a routine use in terminal cleaning procedures. Therefore, additional research under real-life conditions is needed to confirm effectiveness against a wide variety of pathogens as well as for various environmental conditions and surfaces.

## Data Availability Statement

The original contributions presented in the study are included in the article/supplementary material, further inquiries can be directed to the corresponding author.

## Author Contributions

BK and JK conceived and planned the experiments. BK and GF carried out the experiments. BK, GF, EK, CB, and JK contributed to the experiments and the interpretation of the results. BK and JK performed the statistical analyses. BK, GF, and JK wrote the manuscript in consultation with EK and CB. All authors discussed the results and critically revised and approved the final version of manuscript.

## Conflict of Interest

BK and JK received a travel grant from INFUSER Germany GmbH, Mannheim, Deutschland. The remaining authors declare that the research was conducted in the absence of any commercial or financial relationships that could be construed as a potential conflict of interest.
